# Associations of the TyG index, remnant cholesterol levels, and the severity of coronary artery disease: findings from a retrospective study

**DOI:** 10.3389/fcvm.2026.1786243

**Published:** 2026-06-19

**Authors:** Xi Meng, Yibo Duanmu, Xiaoyi Yu, Lijie Liu, Yuhui Cui, Zhimin Chen, Guiyuan Ji, Dantao Zhang, Xingfen Yang, Xilong Liu, Wei Wu

**Affiliations:** 1 Guangdong Provincial Institute of Public Health, Guangdong Provincial Center for Disease Control and Prevention, Guangzhou, China; 2 NMPA Key Laboratory for Safety Evaluation of Cosmetics, Guangdong Provincial Key Laboratory of Tropical Disease Research, School of Public Health, Southern Medical University, Guangzhou, China; 3 Department of Medical Imaging Center, Nanfang Hospital, Southern Medical University, Guangzhou, China

**Keywords:** insulin resistance, mediation analysis, remnant cholesterol, severity of coronary artery disease, triglyceride-glucose index

## Abstract

**Background:**

This study aimed to investigate whether the triglyceride-glucose (TyG) index mediates the association between remnant cholesterol (RC) and coronary artery disease (CAD) severity and to evaluate their combined association with multi-vessel CAD.

**Methods:**

In this retrospective study, anonymized data from CAD patients undergoing coronary computed tomography angiography (CCTA) or coronary angiography (CAG) between 2019 and 2022 were analyzed**.** Patients were classified into single-vessel or multi-vessel disease groups. Insulin resistance was estimated using the TyG index, and RC was calculated. Multivariate logistic regression, restricted cubic spline (RCS) modeling, and mediation analysis were utilized to explore the relationships among the TyG index, RC, and CAD severity.

**Results:**

A total of 592 patients (64.7% male; mean age, 62 years) were included in the analysis. The TyG index was positively correlated with RC (*r* = 0.52, *P* < 0.001). After adjustment for confounding factors, each 1-SD increase in the TyG index was associated with higher odds of multi-vessel CAD (OR = 1.724; 95% CI, 1.323–2.247; *P* < 0.001), and each 1-SD increase in the RC was similarly associated with higher odds of multi-vessel CAD (OR = 1.491; 95% CI, 1.024–2.171; *P* = 0.037). Patients with both high TyG index and high RC had significantly higher odds of multi-vessel CAD (OR = 3.53; 95% CI, 2.040–6.258; *P* < 0.001). Mediation analysis demonstrated that the TyG index mediated 33% of the association between RC and multi-vessel CAD.

**Conclusions:**

The TyG index is positively correlated with RC and is significantly associated with CAD severity. Furthermore, the TyG index partially mediates the association between RC and CAD severity. Combined assessment of the TyG index and RC may improve metabolic risk stratification in patients with CAD.

## Introduction

Coronary artery disease (CAD) remains one of the leading causes of cardiovascular morbidity and mortality worldwide and is characterized by progressive atherosclerotic narrowing or occlusion of the coronary arteries (1, 2). The severity of CAD is strongly influenced by the extent of arterial stenosis, and multi-vessel CAD is associated with worse clinical outcomes, including higher rates of mortality and complications (3). Accurate assessment of CAD severity is essential for determining appropriate therapeutic strategies, but invasive diagnostic techniques, such as coronary angiography (CAG), have limitations including high cost, invasiveness, and risk of complications. This creates an urgent need for non-invasive biomarkers that can reliably predict CAD severity. Multi-vessel CAD reflects a greater overall atherosclerotic burden and is associated with more complex coronary pathology and poorer prognosis.

The pathogenesis of CAD is driven by atherosclerosis, a complex process involving endothelial dysfunction, inflammation, oxidative stress, lipid metabolism disturbances, and thrombosis (4). Insulin resistance, a metabolic abnormality closely associated with cardiometabolic disorders, has emerged as an important contributor to CAD development and progression (5–7). In the setting of systemic insulin resistance, dyslipidemia commonly occurs and is characterized by elevated triglyceride-rich lipoproteins and remnant cholesterol (RC), both of which are implicated in the progression of atherosclerosis (8–10). RC, primarily derived from triglyceride-rich lipoproteins, can penetrate the endothelial barrier and accumulate within arterial walls, thereby promoting lipid deposition, vascular inflammation, and plaque formation (10–12). Despite substantial reductions in low-density lipoprotein cholesterol (LDL-C) achieved with contemporary lipid-lowering therapies, considerable residual cardiovascular risk persists in many patients (13), potentially owing to elevated RC levels. Emerging evidence suggests that the combined effects of insulin resistance and remnant lipoprotein metabolism may contribute substantially to diffuse coronary atherosclerosis and multi-vessel CAD (14, 15). However, the interaction between metabolic dysfunction and remnant lipoprotein metabolism in determining CAD severity remains insufficiently understood.

The triglyceride-glucose (TyG) index, a simple and reliable marker of insulin resistance (16), has demonstrated strong correlations with both the hypoglycemic-hyperinsulin clamp test and the Homeostasis Model Assessment of Insulin Resistance (HOMA-IR) (17). Previous studies have shown that elevated TyG index levels are associated with an increased risk of coronary artery stenosis and greater CAD burden (18–21). In parallel, elevated RC levels have also been associated with coronary artery calcification, plaque burden, and adverse cardiovascular outcomes (22–24). Given the close interrelationship between insulin resistance and remnant lipoprotein metabolism in atherosclerosis, clarifying the interplay among the TyG index, RC, and CAD severity may provide important insights into the metabolic mechanisms underlying severe coronary lesions.

Emerging evidence further suggests that RC is not only an atherogenic lipid parameter but is also closely associated with metabolic dysfunction and insulin resistance (25). Remnant lipoprotein particles may contribute to ectopic lipid accumulation, chronic low-grade inflammation, oxidative stress, and impaired insulin signaling, thereby promoting the development of insulin resistance (26). As a reliable surrogate marker of insulin resistance, the TyG index has been associated with endothelial dysfunction, vascular inflammation, diffuse atherosclerosis, and adverse cardiovascular outcomes. Therefore, it is biologically plausible that the TyG index may partially mediate the association between RC and CAD severity (27). However, whether insulin resistance acts as a potential intermediary pathway linking RC to the severity of coronary atherosclerosis, particularly multi-vessel CAD, remains unclear.

This study aims to provide deeper insights into the combined effects of insulin resistance and RC on CAD severity and to examine whether the TyG index mediates the relationship between RC and multi-vessel CAD. This will improve our understanding of how disturbances in lipid and glucose metabolism contribute to CAD severity and guide the development of more effective clinical intervention strategies.

## Methods

### Study population

This retrospective study initially screened 1,247 patients diagnosed with CAD who underwent coronary computed tomography angiography (CCTA) or coronary angiography (CAG) at Nanfang Hospital of Southern Medical University between January 2019 and December 2023. Participants were excluded according to the following criteria: (1) aged less than 18 years or older than 75 years; (2) missing data on triglycerides (TG), fasting plasma glucose (FPG), total cholesterol (TC), low-density lipoprotein cholesterol (LDL-C), or high-density lipoprotein cholesterol (HDL-C); (3) prior coronary artery bypass grafting (CABG) or percutaneous coronary intervention (PCI); (4) presence of critical arrhythmia, cardiomyopathy, valvular heart disease, congenital heart anomalies, or heart failure; (5) with severe cardiac dysfunction with left ventricular ejection fraction (LVEF) below 30%; (6) with severe hepatic or renal dysfunction, defined as alanine aminotransferase [ALT] and/or aspartate aminotransferase [AST] levels exceeding three times the upper normal or estimated glomerular filtration rate [eGFR] less than 30 mL/min/1.73 m²; (7) with severe chronic obstructive pulmonary disease or asthma; (8) with active infection, thyroid disease, hematological disorders, autoimmune disease, or malignancies; (9) pregnant or lactation; and (10) poor-quality imaging data or severe imaging artifacts. After applying these criteria, a total of 592 patients were included in the final analysis, as illustrated in Supplementary Figure S1.

This study was approved by the Ethics Committee of Nanfang Hospital of Southern Medical University (NFEC–2023–154) and conducted in accordance with the Declaration of Helsinki. Given the retrospective design and use of anonymized clinical data, the requirement for written informed consent was waived by the Ethics Committee. All identifiable personal information was removed prior to data collection and analysis to ensure patient confidentiality and data privacy.

## Data collection

### Clinical and demographic data

Demographic and clinical data were obtained from electronic medical records, including age, gender, height, weight, and the calculated body mass index (BMI), along with systolic blood pressure (SBP), diastolic blood pressure (DBP), smoking status, alcohol use, and medical history (hypertension, diabetes mellitus, stroke, hyperlipidemia, etc.). Information on medication use, including antihypertensive drugs, antidiabetic medications, and antiplatelet agents, was also recorded.

### Biochemical measurements

Blood samples were collected after an overnight fast of at least 8 h for biochemical analysis. The following parameters were assessed using a fully automated biochemical analyzer: white blood cell count (WBC), lymphocyte count, neutrophil count, monocyte count, hemoglobin, alanine aminotransferase (ALT), aspartate aminotransferase (AST), serum creatinine (SCr), uric acid (SUA), C-reactive protein (CRP), triglycerides (TG), total cholesterol (TC), low-density lipoprotein cholesterol (LDL-C), high-density lipoprotein cholesterol (HDL-C), fasting plasma glucose (FPG), and glycated hemoglobin (HbA1c). The left ventricular ejection fraction (LVEF) was assessed by echocardiography. The BMI was calculated as weight (kg) divided by height squared (m²), and the estimated glomerular filtration rate (eGFR) was calculated using the Chronic Kidney Disease Epidemiology Collaboration (CKD-EPI) equation. The TyG index was calculated using the formula: TyG index = Ln [TG (mg/dL) × FPG (mg/dL)/2]. Remnant cholesterol (RC) was calculated by subtracting HDL-C and LDL-C from TC, using a well-established lipid profiling method (8).

### Outcome definition

The presence of CAD was defined as greater than 50% stenosis in at least one coronary artery, as determined by CAG or CCTA, according to the American College of Cardiology and American Heart Association guidelines (28). Patients were categorized as having single-vessel CAD if only one coronary artery was involved and multi-vessel CAD if two or more arteries exhibited ≥50% stenosis.

### Statistical analysis

Descriptive statistics were used to summarize baseline characteristics. Continuous variables were expressed as means ± standard deviation (SD) for normally distributed data, and as medians (P25, P75) for non-normally distributed variables. Comparisons between groups were performed using *t*-tests or the Mann–Whitney *U*-test, depending on the distribution of the data. Categorical variables were reported as frequencies and percentages and compared using chi-square tests or Fisher's exact tests.

The Least Absolute Shrinkage and Selection Operator (LASSO) regression was employed to identify significant predictors of multi-vessel CAD, addressing collinearity issues among variables. By incorporating an L1 regularization term (absolute value penalty term) into the ordinary least squares regression, LASSO regression effectively reduces certain coefficients to near zero, thereby facilitating the identification of the most significant features and enhancing the model's generalization capability.

Multivariate logistic regression models were used to calculate odds ratios (ORs) and 95% confidence intervals (CIs) for the association between the TyG index, RC, and CAD severity. Three models were applied: Model 1 (unadjusted), Model 2 (adjusted for age and gender), and Model 3 (further adjusted for additional variables identified by LASSO regression). The relationship between TyG and RC was explored using Spearman's rank correlation.

The restricted cubic spline (RCS) regression was used to assess potential nonlinear dose-response relationships. To further assess the additive interaction with CAD severity, we employed the receiver operating characteristic (ROC) curve to ascertain the optimal cut-off value. Patients were subsequently categorized into four distinct groups based on variations in the TyG index and RC levels: the low-TyG index/low-RC level group, the low-TyG index/high-RC level group, the high-TyG index/low-RC level group, and the high-TyG index/high-RC level group. The low-TyG index/low-RC level group served as the reference category. Additionally, we refined and categorized the TyG index and RC into a three-group system, integrating them into a multiple logistic regression model for comprehensive analysis. The Synergy Index (SI) was utilized to evaluate interaction effects, where an SI of 1 denotes no interaction, an SI greater than 1 indicates synergistic effects, and an SI less than 1 signifies weaker combined effects.

Subgroup analyses were performed to investigate whether the potential associations between the TyG index and RC with CAD severity were influenced by demographic and clinical characteristics. To examine whether the TyG index mediates the relationship between RC and CAD severity, we employed the “Mediation” package in R and constructed a directed acyclic graph (DAG) to visualize the model. The mediation analysis was performed using 1,000 bootstrap samples. All statistical evaluations were executed using R Foundation for Statistical Computing (version 4.4.1, R Foundation for Statistical Computing, Vienna, Austria), and a two-sided *P*-value < 0.05 was considered statistically significant.

## Results

### Population characteristics

A total of 592 patients with CAD were included in the study, consisting of 263 patients with single-vessel CAD and 329 patients with multi-vessel CAD. The median age was 61 years [interquartile range (IQR): 56–67 years] in the single-vessel CAD group and 64 years (IQR, 56–69 years) in the multi-vessel CAD group. Detailed demographic and clinical characteristics are summarized in Table 1. Compared with patients with single-vessel CAD, those with multi-vessel CAD were generally older, more likely to be male, and had higher proportions of smoking, alcohol consumption, and diabetes mellitus. In addition, patients with multi-vessel CAD were more frequently treated with oral hypoglycemic agents and antiplatelet therapy. Additionally, the multi-vessel CAD group had significantly higher levels of monocytes, albumin, TC, FPG, HbA1c, TyG index, and RC, as well as lower LVEF, compared to the single-vessel CAD group.

**Table 1:** Baseline characteristics of the patients stratified by the CAD severity.

	All subjects	Single-vessel CAD	Multi-vessel CAD	*P* value
No.	(*N* = 592)	(*N* = 263)	(*N* = 329)	
Age, years	62 (56, 68)	61 (56, 67)	64 (56, 69)	0.08
Gender, *n* (%)				
Male	383 (64.7)	148 (56.3)	235 (71.4)	**<0.001**
Female	209 (35.3)	115 (43.7)	94 (28.6)	
BMI, kg/m^2^	24.7 (22.6, 27.1)	24.8 (22.6, 27.1)	24.7 (22.5, 27.1)	0.75
SBP, mmHg	129.0 (122.0, 135.0)	130.0 (123.0, 135.0)	128.0 (122.0, 134.0)	0.11
DBP, mmHg	78.0 (72.0, 83.0)	78.0 (72.0, 83.0)	78.0 (72.0, 83.0)	0.98
HR, bpm	73.0 (66.0, 81.0)	72.0 (66.0, 80.0)	73.0 (66.0, 81.0)	0.58
WBC, *10^9^/L	6.8 (5.8, 8.1)	6.8 (5.7, 8.1)	6.8 (5.8, 8.1)	0.66
Lymphocyte, *10^9^/L	2.0 (1.6, 2.4)	2.0 (1.5, 2.4)	2.0 (1.6, 2.4)	0.87
Neutrophil, *10^9^/L	4.1 (3.2, 5.0)	4.1 (3.2, 5.0)	4.2 (3.3, 5.0)	0.60
Monocyte, *10^9^/L	0.5 (0.4, 0.6)	0.5 (0.4, 0.6)	0.7 (0.6, 0.8)	**0.013**
Hemoglobin, g/L	138.3 ± 15.4	138.0 ± 14.3	138.5 ± 16.2	0.70
ALT, U/L	19.0 (14.0, 27.0)	20.0 (15.0, 27.0)	18.0 (13.0, 27.0)	0.12
AST, U/L	19.0 (16.0, 24.0)	20.0 (16.0, 24.5)	19.0 (15.0, 23.0)	0.09
Albumin, g/L	43.5 (41.0, 45.9)	43.2 (40.8, 45.7)	43.9 (41.2, 46.2)	**0.031**
Globulin, g/L	26.3 (23.8, 29.0)	26.6 (24.0, 29.6)	25.9 (23.7, 28.8)	0.19
SCr, μmol/L	78.0 (65.0, 90.0)	78.0 (63.5, 89.0)	78.0 (67.0, 90.0)	0.16
SUA, μmol/L	349.5 (291.8, 425.0)	341.0 (286.5, 421.0)	356.0 (294.0, 426.0)	0.13
eGFR, mL/min/1.73 m^2^	87.0 (75.3, 96.4)	87.6 (74.7, 96.8)	86.5 (75.7, 96.1)	0.53
CRP, mg/L	1.5 (0.7, 4.1)	1.3 (0.7, 3.9)	1.7 (0.7, 4.1)	0.14
TG, mmol/L	1.6 (1.2, 2.7)	1.7 (1.1, 2.6)	1.6 (1.2, 2.7)	0.93
TC, mmol/L	4.8 (3.9, 5.6)	4.6 (3.9, 5.5)	4.8 (4.1, 5.7)	**0.034**
HDL-C, mmol/L	1.1 (0.9, 1.3)	1.1 (0.9, 1.3)	1.1 (0.9, 1.3)	0.79
LDL-C, mmol/L	2.9 (2.3, 3.5)	2.8 (2.2, 3.5)	2.9 (2.4, 3.5)	0.059
RC, mmol/L	0.6 (0.4, 0.9)	0.6 (0.4, 0.8)	0.7 (0.4, 0.9)	**0.029**
FPG, mmol/L	5.9 (5.2, 7.3)	5.7 (5.1, 6.6)	6.1 (5.3, 8.2)	**<0.001**
HbA1c, %	6.2 (5.9, 6.9)	6.2 (5.8, 6.7)	6.3 (5.9, 7.4)	**0.001**
TyG index	8.9 (8.6, 9.6)	8.9 (8.6, 9.4)	9.0 (8.6, 9.7)	**0.015**
LVEF, %	63.0 (59.6, 66.0)	63.2 (60.0, 66.9)	62.5 (59.0, 65.6)	**0.020**
Smoking status, *n* (%)	286 (48.3)	105 (39.9)	181 (55.0)	**<0.001**
Drinking status, *n* (%)	236 (39.9)	91 (34.6)	145 (44.1)	**0.024**
Hypertension, *n* (%)	452 (76.4)	195 (74.1)	257 (78.1)	0.30
Diabetes mellitus, *n* (%)	256 (43.2)	96 (36.5)	160 (48.6)	**0.004**
Hyperlipidemia, *n* (%)	352 (59.5)	165 (62.7)	187 (56.8)	0.17
Stroke, *n* (%)	115 (19.4)	45 (17.1)	70 (21.3)	0.24
Family History of CVD, *n* (%)	203 (34.3)	93 (35.4)	110 (33.4)	0.69
Anti-hypertensive drugs, *n* (%)	407 (68.8)	178 (67.7)	229 (69.6)	0.68
Oral antidiabetic drugs, *n* (%)	250 (42.2)	91 (34.6)	159 (48.3)	**0.001**
Antiplatelet drugs, *n* (%)	332 (56.1)	106 (40.3)	226 (68.7)	**<0.001**

CAD, coronary artery disease; BMI, body mass index; SBP, systolic blood pressure; DBP, diastolic blood pressure; HR, heart rate; WBC, white blood cells; ALT, alanine aminotransferase; AST, aspartate aminotransferase; SCr, serum creatinine; SUA, serum uric acid; eGFR, estimated glomerular filtration rate; CRP, C-reactive protein; TG, triglycerides; TC, total cholesterol; HDL-C, high-density lipoprotein-C; LDL-C, low-density lipoprotein-C; RC, remnant cholesterol; FPG, fasting plasma glucose; HbAlc, glycated hemoglobin; TyG index, triglyceride-glucose index; LVEF, left ventricular ejection fraction; SD, standard deviation. Data are presented as mean ± SD, median (interquartile range), or *n* (%).

Bold values indicates statistical significance at *P* < 0.05.

### Association between RC and TyG index

A significant positive correlation was observed between the TyG index and RC levels (Supplementary Figure S2; Spearman's *r* = 0.52; *P* < 0.001).

### Results of the LASSO regression

**Figure 1:**
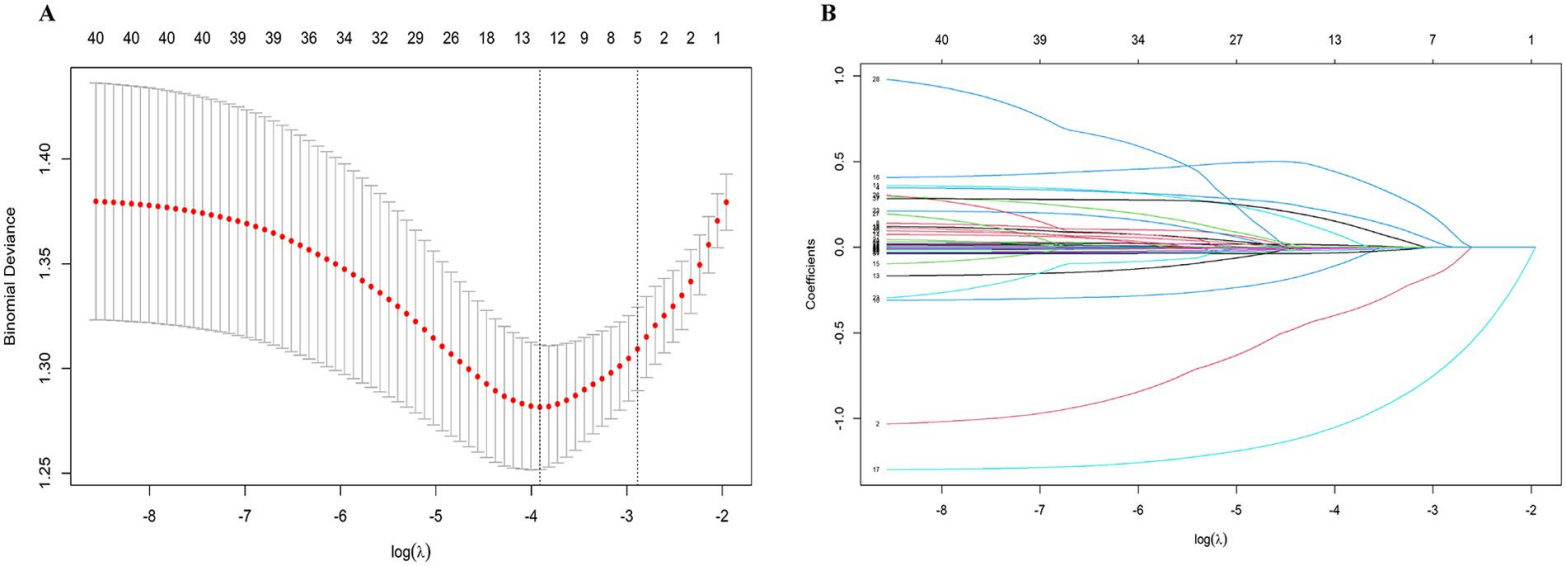
The LASSO regression was utilized to identify key predictors related to the severity of CAD. [**(A)** The process of screening the regularization parameter *λ*; **(B)** Changes of 40 variables with changes in the regularization parameter *λ*].

LASSO regression analysis with 10-fold cross-validation was performed to identify variables associated with CAD severity. The maximum number of iterations was set to 1,000 to ensure model convergence. As shown in Figure 1A, the minimum cross-validation error was achieved at *λ*_min = 0.020, whereas the more conservative *λ*_1se = 0.056 resulted in a 3.3% increase in the cross-validation error. Therefore, *λ*_min was selected for variable selection. Using this criterion, 10 variables with non-zero coefficients were identified as key indicators associated with CAD severity, including age, gender, smoking status, SBP, history of hyperlipidemia, stroke, antiplatelet drug usage, AST levels, albumin levels, and LVEF (Figures 1A,B). This approach balanced model stability and predictive performance while reducing the risk of overfitting.

### Association of TyG index and RC levels with CAD severity

Multivariate logistic regression analysis demonstrated that each 1-SD increase in the TyG index was associated with higher odds of multi-vessel CAD (OR = 1.724; 95% CI, 1.323–2.247; *P* < 0.001) after adjustment for variables identified through LASSO regression, including age, gender, smoking status, SBP, history of hyperlipidemia, stroke, use of antiplatelet drugs, AST levels, albumin levels, and LVEF. In the categorical analysis, patients in the highest TyG tertile (T3) had significantly higher odds of multi-vessel CAD compared with those in the lowest tertile (T1) (OR = 1.696; 95% CI, 1.091–2.638; *P* = 0.02). Similarly, each 1-SD increase in RC was associated with higher odds of multi-vessel CAD (OR = 1.491; 95% CI, 1.024–2.171; *P* = 0.037). In categorical analysis, patients in the highest RC tertile (R3) had significantly higher odds of multi-vessel CAD compared with those in the lowest tertile (R1) (OR = 1.912; 95% CI, 1.226–2.980; *P* = 0.004) (Table 2).

**Table 2:** Association of the TyG index and RC levels with CAD severity.

Variables	Model 1		Model 2		Model 3	
	OR (95% CI)	P-value	OR (95% CI)	P-value	OR (95% CI)	P-value
TyG index (per SD) TyG index (tertiles)	1.499 (1.185–1.896)	0.001	1.603 (1.259–2.043)	<0.001	1.724 (1.323–2.247)	<0.001
T1 group	*Reference*		*Reference*		*Reference*	
T2 group	0.917 (0.617–1.361)	0.67	0.975 (0.651–1.459)	0.90	1.126 (0.731–1.733)	0.59
T3 group	1.416 (0.950–2.110)	0.09	1.546 (1.027–2.328)	0.037	1.696 (1.091–2.638)	0.02
RC level (per SD) RC level (tertiles)	1.355 (0.982–1.870)	0.06	1.398 (1.009–1.937)	0.044	1.491 (1.024–2.171)	0.037
R1 group	*Reference*		*Reference*		*Reference*	
R2 group	1.135 (0.767–1.680)	0.53	1.084 (0.727–1.617)	0.69	1.114 (0.726–1.710)	0.62
R3 group	1.527 (1.024–2.277)	0.038	1.597 (1.062–2.403)	0.03	1.912 (1.226–2.980)	0.004

Model 1: unadjusted; Model 2: Adjusted for age, gender; Model 3: Adjusted for age, gender, smoking status, SBP, hyperlipemia, stroke, AST, albumin, antiplatelet drugs and LVEF.

TyG index, triglyceride-glucose index; RC, remnant cholesterol; CAD, coronary artery disease; SD, standard deviation; OR, odds ratios; CI, confidence interval; SBP, systolic blood pressure; AST, aspartate aminotransferase; LVEF, Left ventricular ejection fraction.

**Figure 2:**
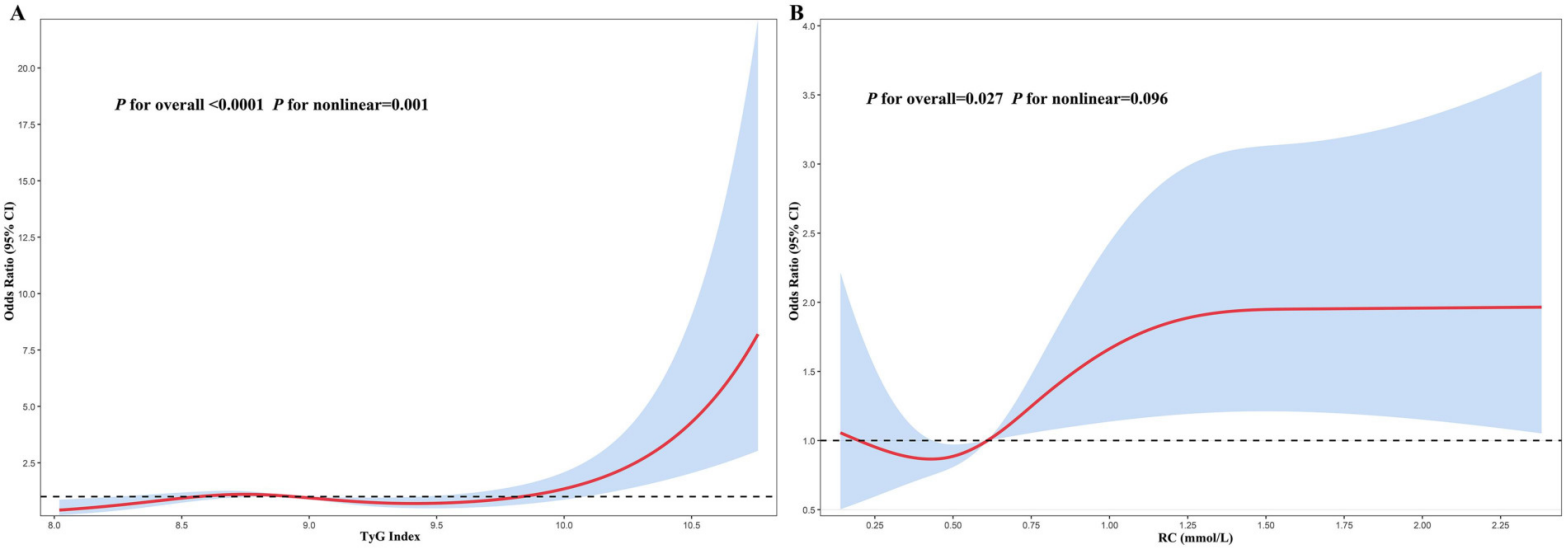
RCS linear regression analysis of the relationship between the TyG index and RC levels with the severity of CAD. [**(A)** RCS linear regression analysis of the TyG index and severity of CAD; (**B)** RCS linear regression analysis of RC levels and severity of CAD). All models were adjusted for age, gender, smoking status, SBP, hyperlipemia, stroke, AST, albumin, antiplatelet drugs and LVEF. Data were fitted by multivariate logistic regression model. Solid lines indicate ORs, and shadow shapes indicate 95% CIs. TyG index, triglyceride-glucose index; RC, remnant cholesterol; CAD, coronary artery disease; OR, odds ratios; CI, confidence interval; SBP, systolic blood pressure; AST, aspartate aminotransferase; LVEF, Left ventricular ejection fraction.

Furthermore, restricted cubic spline (RCS) regression models were used to characterize the dose-response associations between the TyG index, RC levels, and CAD severity (Figures 2A,B). After adjustment for potential confounders, a significant nonlinear association was observed between the TyG index and multi-vessel CAD (*P*_for nonlinearity_ = 0.001). Notably, the odds of multi-vessel CAD increased sharply when the TyG index exceeded 9.838. In contrast, the association between RC levels and multi-vessel CAD remained approximately linear (*P*_for nonlinearity_ = 0.096). Although a nonlinear association was identified for the TyG index, the primary logistic regression models presented in Table 2 were retained as linear estimates because they provide clinically interpretable overall effect sizes and facilitate comparison across continuous and categorical exposure models. The RCS analysis was additionally performed as an exploratory approach to visualize the dose-response pattern and identify potential threshold effects.

### Interaction and joint analysis

After categorizing the TyG index and RC levels according to the optimal cutoff values and adjusting for covariates, patients with both high TyG index and high RC levels exhibited significantly higher odds of multi-vessel CAD (OR = 3.53; 95% CI: 2.040–6.258; *P* < 0.001), as shown in Table 3. Similar findings were observed in the grouped recombination analysis. Specifically, patients in the highest tertiles of both the TyG index (T3) and RC (R3) had significantly higher odds of multi-vessel CAD compared with those in the lowest tertiles (T1 and R1) (OR = 2.45; 95% CI: 1.36–4.48; *P* = 0.003) (Supplementary Table S1). However, no significant additive interaction was observed (Synergy Index, 1.04; 95% CI, 0.367–1.723).

**Table 3:** Joint analysis of the association of the TyG index and RC levels with CAD severity by optimal cutoff values divide into groups.

Groups	Model 1	*P*-value	Model 2	*P*-value	Model 3	
	OR (95% CI)		OR (95% CI)		OR (95% CI)	*P*-value
Low TyG index/Low RC level	Reference		Reference		Reference	
Low TyG index/High RC level	1.74 (1.185–2.553)	0.005	1.76 (1.193–2.613)	0.005	2.01 (1.315–3.088)	0.001
High TyG index/Low RC level	2.76 (1.311–6.239)	0.01	2.87 (1.339–6.582)	0.009	3.12 (1.392–7.450)	0.007
High TyG index/High RC level	2.88 (1.749–4.851)	<0.001	3.14 (1.882–5.355)	<0.001	3.53 (2.040–6.258)	<0.001

Model 1: unadjusted; Model 2: Adjusted for age, gender; Model 3: Adjusted for age, gender, smoking status, SBP, hyperlipemia, stroke, AST, albumin, antiplatelet drugs and LVEF.

TyG index, triglyceride-glucose index; RC, remnant cholesterol; CAD, coronary artery disease; SD, standard deviation; OR, odds ratios; CI, confidence interval; SBP, systolic blood pressure; AST, aspartate aminotransferase; LVEF, Left ventricular ejection fraction.

### Subgroup analysis and mediation analysis

**Figure 3:**
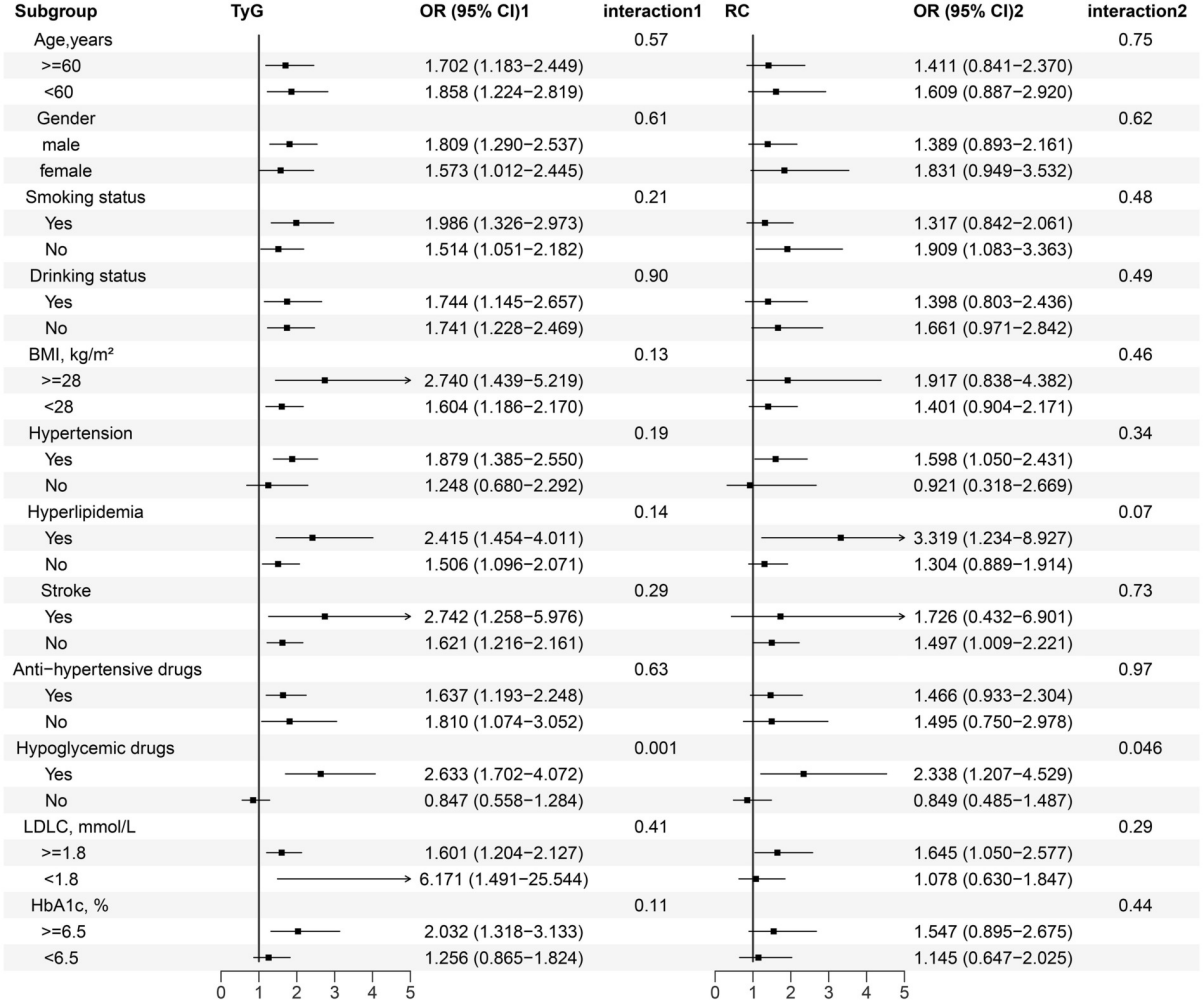
Forest plot for subgroup analysis of the association between the TyG index and RC levels and the severity of CAD. All models were adjusted for age, gender, smoking status, SBP, hyperlipemia, stroke, AST, albumin, antiplatelet drugs and LVEF. TyG index, triglyceride-glucose index; RC, remnant cholesterol; CAD, coronary artery disease; OR, odds ratios; CI, confidence interval; SBP, systolic blood pressure; AST, aspartate aminotransferase; LVEF, Left ventricular ejection fraction.

Subgroup analyses and interaction tests were conducted to evaluate whether the associations of the TyG index and RC with CAD severity differed across clinically relevant strata (Supplementary Table S2). As shown in Figure 3, no significant interactions were observed for age, gender, smoking status, alcohol consumption, BMI, history of hypertension, hyperlipidemia, use of antihypertensive medications, and HbA1c levels (all *P*_for interaction_ > 0.05), suggesting that these variables did not substantially modify the observed associations. However, the use of hypoglycemic medications significantly modified the associations of both the TyG index and RC levels with multi-vessel CAD (both *P*_for interaction_ < 0.05). Among patients using hypoglycemic medications, each 1-SD increase in the TyG index was associated with significantly higher odds of multi-vessel CAD (OR = 2.633; 95% CI, 1.702–4.072; *P* < 0.001). Similarly, elevated RC levels were associated with increased odds of multi-vessel CAD in this subgroup (OR = 2.338; 95% CI, 1.207–4.529; *P* = 0.046).

**Figure 4:**
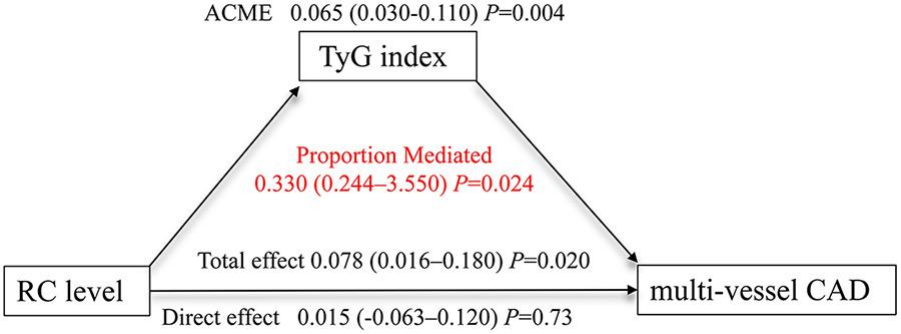
Mediated effects by the TyG index on the associations of RC levels with multi-vessel CAD. All models were adjusted for age, gender, smoking status, SBP, hyperlipemia, stroke, AST, albumin, antiplatelet drugs and LVEF. TyG index, triglyceride-glucose index; RC, remnant cholesterol; CAD, coronary artery disease; OR, odds ratios; CI, confidence interval; SBP, systolic blood pressure; AST, aspartate aminotransferase; LVEF, Left ventricular ejection fraction.

Mediation analysis further demonstrated that the TyG index significantly mediated 33% of the association between RC levels and multi-vessel CAD in the fully adjusted model (*P* = 0.024) (Figure 4). In contrast, RC mediated only 5% of the association between the TyG index and multi-vessel CAD, and this indirect effect did not reach statistical significance (*P* = 0.72) (Supplementary Figure S3). Detailed mediation analysis results are presented in Supplementary Table S3.

## Discussion

This study provides further insights into the interplay between insulin resistance and remnant cholesterol metabolism in the progression of severe coronary artery disease (CAD). Several important findings emerged from our analyses. First, both the TyG index and RC levels were independently associated with higher odds of multi-vessel CAD. Second, the TyG index exhibits a nonlinear dose-response relationship with CAD severity, with the risk of multi-vessel CAD increasing sharply when the TyG index exceeds 9.838. Third, the associations between the TyG index and RC levels with CAD severity were significantly modified by the use of hypoglycemic medications. Finally, mediation analysis revealed that the TyG index partially mediated the effect between RC and CAD severity, accounting for 33% of the total effect, whereas no significant additive synergy effect between the TyG index and RC was observed. Collectively, these findings highlight the complex metabolic interaction between insulin resistance and remnant lipoprotein metabolism in patients with multi-vessel CAD.

The TyG index, a reliable biomarker of insulin resistance, has been consistently associated with CAD severity in previous studies. Thai et al. (18) reported that a TyG index exceeding 10 was significantly associated with severe coronary stenosis (>70%), while Tang et al. (29) demonstrated a positive correlation between the TyG index and Gensini score in patients with CAD. In both prediabetic and diabetic populations, elevated TyG index levels have also been linked to a higher prevalence of multi-vessel CAD (19, 20). Furthermore, several studies have demonstrated a significant association between the TyG index and acute coronary syndrome (ACS), suggesting that the TyG index may help identify CAD patients at high cardiovascular risk (30, 31). Our findings are consistent with these observations and further support the value of the TyG index as a clinically accessible biomarker for evaluating coronary lesion burden and metabolic risk in patients with CAD.

Increasing evidence also supports the important role of RC in the development and progression of atherosclerotic cardiovascular disease. Clinical, imaging, and genetic studies have demonstrated significant associations between RC levels and both coronary plaque burden and plaque instability. Notably, Mendelian randomization analyses have suggested a potential causal relationship between elevated RC levels and CAD risk (32). In addition, studies based on the SYNTAX (23) and the Gensini score (24) have shown that elevated RC levels are associated with more severe coronary artery lesions. A prospective study involving 6,544 individuals without atherosclerotic cardiovascular disease (ASCVD) further demonstrated that RC remained significantly associated with coronary artery calcification, even among individuals with well-controlled low-density lipoprotein cholesterol (LDL-C) levels (33). Consistent with these findings, our study demonstrated significantly higher RC levels in patients with multi-vessel CAD, reinforcing the potential importance of RC as a residual lipid-related risk factor in severe coronary atherosclerosis.

An important finding of our study was the identification of a nonlinear association between the TyG index, RC, and multi-vessel CAD. Previous studies have generally reported a linear or dose-dependent relationship between the TyG index and CAD severity (20). In contrast, our analysis identified a significant nonlinear relationship (*P*_for nonlinearity_ = 0.001), where CAD risk increases when the TyG index is below 8.94, decreases between 8.94 and 9.84, and increases again above 9.84. This finding may reflect the complex metabolic and vascular consequences of worsening insulin resistance. Mechanistically, insulin resistance can promote hyperglycemia, oxidative stress, endothelial dysfunction, vascular inflammation, and abnormal lipid metabolism, all of which accelerate atherosclerotic progression (34, 35). Structural endothelial injury and inflammatory cell infiltration may further amplify vascular dysfunction and plaque progression (36). These mechanisms may partly explain the threshold effect observed in our analysis.

By comparison, the association between RC levels and multi-vessel CAD appeared approximately linear in our study. RC-rich lipoproteins can penetrate the arterial wall and carry oxidized phospholipids that promote endothelial dysfunction, inflammation, and plaque formation (37, 38). Differences between our findings and previous studies reporting nonlinear RC associations may be related to variations in statistical modeling strategies, covariate adjustment, study populations, and CAD severity assessment methods. In the present study, variable selection was performed using LASSO regression, which additionally incorporated factors such as AST, albumin levels, and LVEF into the fully adjusted models. Moreover, differences in sample size and population characteristics may also contribute to inconsistencies across studies.

While previous studies have independently examined the associations of the TyG index and RC with CAD severity, our study further explored the potential mechanistic relationship between these factors using mediation analysis. We observed that the TyG index significantly mediated approximately one-third of the association between RC and multi-vessel CAD, suggesting that disturbances in glucose-insulin metabolism may partially transmit the adverse vascular effects of remnant lipoproteins. These findings are biologically plausible because RC-rich particles may contribute to ectopic lipid accumulation, chronic inflammation, oxidative stress, and impaired insulin signaling, thereby promoting systemic insulin resistance and accelerating coronary atherosclerosis.

Importantly, the reverse mediation pathway, in which RC was modeled as the mediator between the TyG index and CAD severity, was not statistically significant. This asymmetry may indicate that insulin resistance represents a more central downstream pathway through which RC contributes to coronary lesion progression, although residual confounding and alternative biological mechanisms cannot be excluded. In addition, no significant additive interaction was observed between the TyG index and RC, suggesting that their combined effects on CAD severity may primarily reflect overlapping metabolic and inflammatory pathways rather than synergistic biological amplification (37).

The studies discussed above suggest that the progression of multi-vessel CAD may be influenced by the combined effects of insulin resistance and remnant lipoprotein metabolism. Several mechanisms may explain the complex relationship among the TyG index, RC, and CAD severity. Insulin resistance impairs glucose metabolism and promotes dyslipidemia, characterized by elevated triglyceride-rich lipoproteins and RC levels (9, 10). Disturbances in glucose and lipid homeostasis further contribute to oxidative stress, all of which accelerate atherosclerosis progression (8, 39). In addition, remnant lipoprotein particles can penetrate the arterial wall and be directly taken up by macrophages without prior oxidative modification (38), thereby promoting foam cell formation and plaques progression (37). Elevated RC levels may also contribute to ectopic lipid accumulation lipotoxicity, impairing insulin signaling and further aggravating insulin resistance (37). Meanwhile, insulin resistance enhances hepatic VLDL production and reduces insulin-mediated glucose uptake, leading to a vicious cycle of metabolic dysfunction (10). Consistent with these mechanisms, our study demonstrated a significant positive correlation between the TyG index and RC levels, further supporting the close metabolic interplay between insulin resistance and remnant cholesterol metabolism in severe CAD.

The interaction between hypoglycemic medication and CAD severity was observed in our subgroup analysis further supports the metabolic contribution to coronary atherosclerosis progression. Glucose-lowering therapies may influence CAD progression through improvement of insulin sensitivity, glycemic control, vascular inflammation, and lipid metabolism. Previous studies have shown that GLP-1 receptor agonists, SGLT2 inhibitors, metformin, and thiazolidinediones exert beneficial metabolic and vascular effects beyond glucose reduction (40–44). These findings suggest that integrated metabolic management may be important in CAD patients with concomitant insulin resistance and elevated RC levels.

Beyond demonstrating the independent associations of the TyG index and RC with multi-vessel CAD, our findings further suggest that insulin resistance may partially mediate the adverse effects of RC on coronary lesion severity. In addition, combined elevation of the TyG index and RC identified a subgroup of patients with substantially increased risk of multi-vessel CAD, highlighting the potential clinical value of integrated metabolic-lipid risk assessment. The nonlinear association between the TyG index and CAD severity further suggests the presence of a metabolic threshold associated with accelerated atherosclerotic burden. Collectively, these findings provide additional insight into the metabolic mechanisms underlying severe CAD and may help improve early risk stratification and individualized prevention strategies in high-risk patients.

Several limitations should be acknowledged. First, owing to the retrospective cross-sectional design, causal relationships among RC, the TyG index, and CAD severity cannot be definitively established. Although mediation analysis was performed to explore potential intermediary pathways, the observed mediation effects reflect statistical associations rather than causality. In addition, residual and unmeasured confounding factors, including lifestyle behaviors, inflammatory status, socioeconomic factors, and genetic susceptibility, may have influenced the observed associations and mediation pathways. Second, this was a single-center study involving hospitalized patients, which may introduce selection bias and limit the generalizability of the findings to broader populations. Third, RC was calculated using the conventional formula (TC−HDL-C−LDL-C) rather than directly measured. Although this method is widely used in clinical studies because of its practicality and cost-effectiveness, measurement error may occur, particularly in individuals with elevated triglyceride levels or very low LDL-C concentrations. Such inaccuracies may reduce estimate precision and attenuate the observed associations. Finally, although the overall sample size was adequate for the primary analyses, subgroup and interaction analyses may have been underpowered in certain smaller strata, particularly medication-defined subgroups. Therefore, the possibility of Type II error cannot be completely excluded.

## Conclusions

The TyG index is closely associated with RC levels, and both are independently associated with multi-vessel CAD severity. As a marker of insulin resistance, the TyG index partially mediates the association between RC and severe CAD. Given their simplicity and wide clinical availability, combined assessment of the TyG index and RC may provide a practical approach for identifying patients at high risk for severe CAD and improving metabolic-cardiovascular risk stratification.

## Data Availability

The original contributions presented in the study are included in the article/Supplementary Material, further inquiries can be directed to the corresponding author/s.
