# Supplementary material for: Associations of the TyG index, remnant cholesterol levels, and the severity of coronary artery disease: findings from a retrospective study
Source: Front Cardiovasc Med. 2026 Jun 19;13:1786243. doi: 10.3389/fcvm.2026.1786243 (PMC13328199; doi:10.3389/fcvm.2026.1786243)
Supplement: Supplementary file 1 [file Datasheet1.docx]

**Supplementary Table S1 Interaction and joint analysis of the association of TyG index and RC levels with CAD severity by grouping variables**

| TyG index | RC levels | OR (95% CI) | P-value |
| --- | --- | --- | --- |
| T1 group | R1 group | Reference |  |
| T1 group | R2 group | 1.29 (0.66–2.53) | 0.46 |
| T1 group | R3 group | 2.68 (1.01–7.61) | 0.054 |
| T2 group | R1 group | 1.18 (0.62–2.26) | 0.62 |
| T2 group | R2 group | 1.21 (0.62–2.36) | 0.57 |
| T2 group | R3 group | 2.33 (1.11–4.98) | 0.026 |
| T3 group | R1 group | 6.35 (2.03–24.57) | 0.003 |
| T3 group | R2 group | 1.15 (0.56–2.35) | 0.70 |
| T3 group | R3 group | 2.45 (1.36–4.48) | 0.003 |
| SI |  | 1.04 (0.367–1.723) | 0.90 |

**Notes:**This model adjusted for for age, gender, smoking status, SBP, hyperlipemia, stroke, AST, albumin, antiplatelet drugs and LVEF.

**Abbreviations:** TyG index, triglyceride-glucose index; RC, remnant cholesterol; CAD, coronary artery disease; OR, odds ratios; CI, confidence interval; SBP, systolic blood pressure; AST, aspartate aminotransferase; LVEF, left ventricular ejection fraction.

**Supplementary Table S2 Subgroup analysis of the associations of the TyG index and RC levels with CAD severity**

| **Subgroups** | **TyG index** | | | **RC Levels** | | |
| --- | --- | --- | --- | --- | --- | --- |
|  | OR(95% CI) | *P* value | *P* _for interaction_ | OR(95% CI) | *P* value | *P* _for interaction_ |
| **Age,years** |  |  | 0.57 |  |  | 0.75 |
| >=60 | 1.702 (1.183–2.449) | 0.004 |  | 1.411 (0.841–2.370) | 0.19 |  |
| <60 | 1.858 (1.224–2.819) | 0.003 |  | 1.609 (0.887–2.920) | 0.12 |  |
| **Gender** |  |  | 0.61 |  |  | 0.62 |
| male | 1.809 (1.290–2.537) | < 0.001 |  | 1.389 (0.893–2.161) | 0.14 |  |
| female | 1.573 (1.012–2.445) | 0.044 |  | 1.831 (0.949–3.532) | 0.071 |  |
| **Smoking status** |  |  | 0.21 |  |  | 0.48 |
| Yes | 1.986 (1.326–2.973) | < 0.001 |  | 1.317 (0.842–2.061) | 0.23 |  |
| No | 1.514 (1.051–2.182) | 0.026 |  | 1.909 (1.083–3.363) | 0.025 |  |
| **Drinking status** |  |  | 0.90 |  |  | 0.49 |
| Yes | 1.744 (1.145–2.657) | 0.01 |  | 1.398 (0.803–2.436) | 0.24 |  |
| No | 1.741 (1.228–2.469) | 0.002 |  | 1.661 (0.971–2.842) | 0.064 |  |
| **BMI, kg/m^2^** |  |  | 0.13 |  |  | 0.46 |
| >=28 | 2.740 (1.439–5.219) | 0.002 |  | 1.917 (0.838–4.382) | 0.12 |  |
| <28 | 1.604 (1.186–2.170) | 0.002 |  | 1.401 (0.904–2.171) | 0.13 |  |
| **Hypertension** |  |  | 0.19 |  |  | 0.34 |
| Yes | 1.879 (1.385–2.550) | < 0.001 |  | 1.598 (1.050–2.431) | 0.029 |  |
| No | 1.248 (0.680–2.292) | 0.47 |  | 0.921 (0.318–2.669) | 0.88 |  |
| **Diabetes mellitus** |  |  | **<0.001** |  |  | 0.062 |
| Yes | 2.786 (1.762–4.406) | < 0.001 |  | 2.140 (1.106–4.141) | 0.024 |  |
| No | 0.914 (0.616–1.358) | 0.66 |  | 0.912 (0.526–1.580) | 0.74 |  |
| **Hyperlipidemia** |  |  | 0.14 |  |  | 0.072 |
| Yes | 2.415 (1.454–4.011) | < 0.001 |  | 3.319 (1.234–8.927) | 0.017 |  |
| No | 1.506 (1.096–2.071) | 0.012 |  | 1.304 (0.889–1.914) | 0.18 |  |
| **Stroke** |  |  | 0.29 |  |  | 0.73 |
| Yes | 2.742 (1.258–5.976) | 0.011 |  | 1.726 (0.432–6.901) | 0.44 |  |
| No | 1.621 (1.216–2.161) | < 0.001 |  | 1.497 (1.009–2.221) | 0.045 |  |
| **Anti-hypertensive drugs** |  |  | 0.63 |  |  | 0.97 |
| Yes | 1.637 (1.193–2.248) | 0.002 |  | 1.466 (0.933–2.304) | 0.10 |  |
| No | 1.810 (1.074–3.052) | 0.026 |  | 1.495 (0.750–2.978) | 0.25 |  |
| **Hypoglycemic drugs** |  |  | **0.001** |  |  | **0.046** |
| Yes | 2.633 (1.702–4.072) | < 0.001 |  | 2.338 (1.207–4.529) | 0.012 |  |
| No | 0.847 (0.558–1.284) | 0.43 |  | 0.849 (0.485–1.487) | 0.57 |  |
| **HbA1c, %** |  |  | 0.11 |  |  | 0.44 |
| >=6.5 | 2.032 (1.318–3.133) | 0.001 |  | 1.547 (0.895–2.675) | 0.12 |  |
| <6.5 | 1.256 (0.865–1.824) | 0.23 |  | 1.145 (0.647–2.025) | 0.64 |  |

**Notes:**This model adjusted for for age, gender, smoking status, SBP, hyperlipemia, stroke, AST, albumin, dual-antiplatelet drugs and LVEF.

**Abbreviations:** TyG index, triglyceride-glucose index; RC, remnant cholesterol; CAD, coronary artery disease; BMI, body mass index; OR, odds ratios; CI, confidence interval; SBP, systolic blood pressure; AST, aspartate aminotransferase; LVEF, Left ventricular ejection fraction.

**Supplementary Table S3 Mediation effect of the associations between TyG index and RC levels with multi-vessel CAD.**

|  | Estimate | OR(95% CI) | *P* value |
| --- | --- | --- | --- |
| **RC -> TyG index->** **multi-vessel CAD** |  |  |  |
| ACME | 0.065 | 0.065 (0.030–0.110) | 0.004 |
| ADE | 0.015 | 0.015 (-0.063–0.120) | 0.73 |
| Total effect | 0.078 | 0.078 (0.016–0.180) | 0.020 |
| Proportion mediated | 0.330 | 0.330 (0.244–3.550) | 0.024 |
| **TyG index -> RC -> multi-vessel CAD** |  |  |  |
| ACME | 0.001 | 0.001 (-0.003–0.010) | 0.72 |
| ADE | 0.008 | 0.008 (0.001–0.030) | <0.001 |
| Total effect | 0.009 | 0.009 (0.001–0.030) | <0.001 |
| Proportion mediated | 0.050 | 0.050 (-0.240–0.470) | 0.72 |

**Notes:**This model adjusted for for age, gender, smoking status, SBP, hyperlipemia, stroke, AST, albumin, dual-antiplatelet drugs and LVEF.

**Abbreviations:** TyG index, triglyceride-glucose index; RC, remnant cholesterol; CAD, coronary artery disease; ACME, average causal mediation effect; ADE, average direct effect;OR, odds ratios; CI, confidence interval; SBP, systolic blood pressure; AST, aspartate aminotransferase; LVEF, Left ventricular ejection fraction.
