# Supplementary material for: Associations of the TyG index, remnant cholesterol levels, and the severity of coronary artery disease: findings from a retrospective study
Source: Front Cardiovasc Med. 2026 Jun 19;13:1786243. doi: 10.3389/fcvm.2026.1786243 (PMC13328199; doi:10.3389/fcvm.2026.1786243)
Supplement: Supplementary file 2 [file Datasheet2.docx]

**
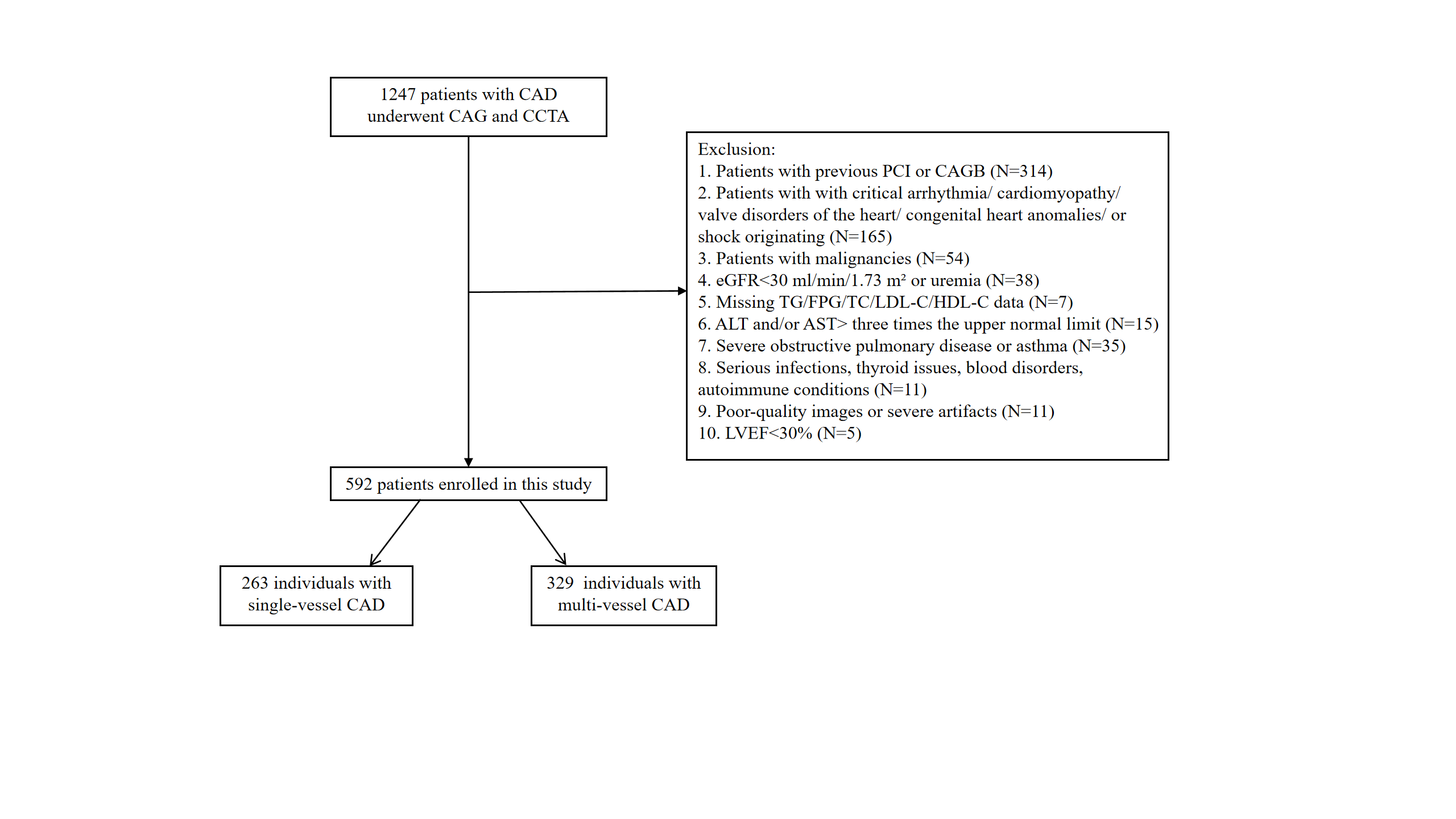
**

**Supplementary Figure S1** Flowchart of study patients. CAD, coronary artery disease; CAG, invasive coronary angiography; CCTA, coronary computed tomography angiography; PCI, percutaneous coronary intervention; CABG, coronary artery bypass grafting; eGFR, estimated glomerular filtration rate; TG, triglycerides; FPG, fasting plasma glucose; TC, total cholesterol; LDL-C, low-density lipoprotein cholesterol; HDL-C, high-density lipoprotein cholesterol; LDL-C, low-density lipoprotein cholesterol; HDL-C, high-density lipoprotein cholesterol; LVEF, left ventricular ejection fraction.


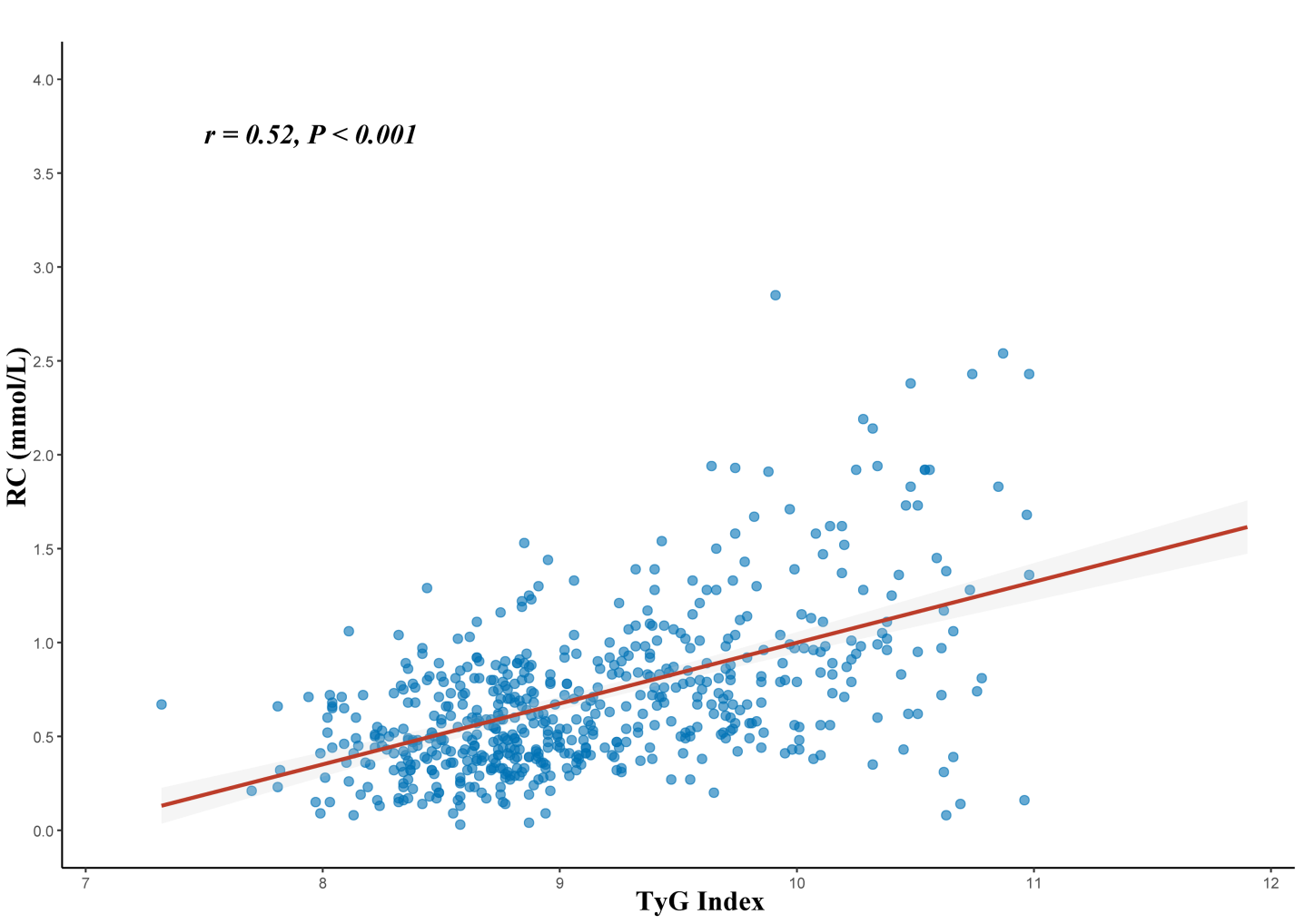


**Supplementary Figure S2** Spearman correlation analysis of TyG index and RC levels. TyG, triglyceride-glucose index; RC, remnant cholesterol.


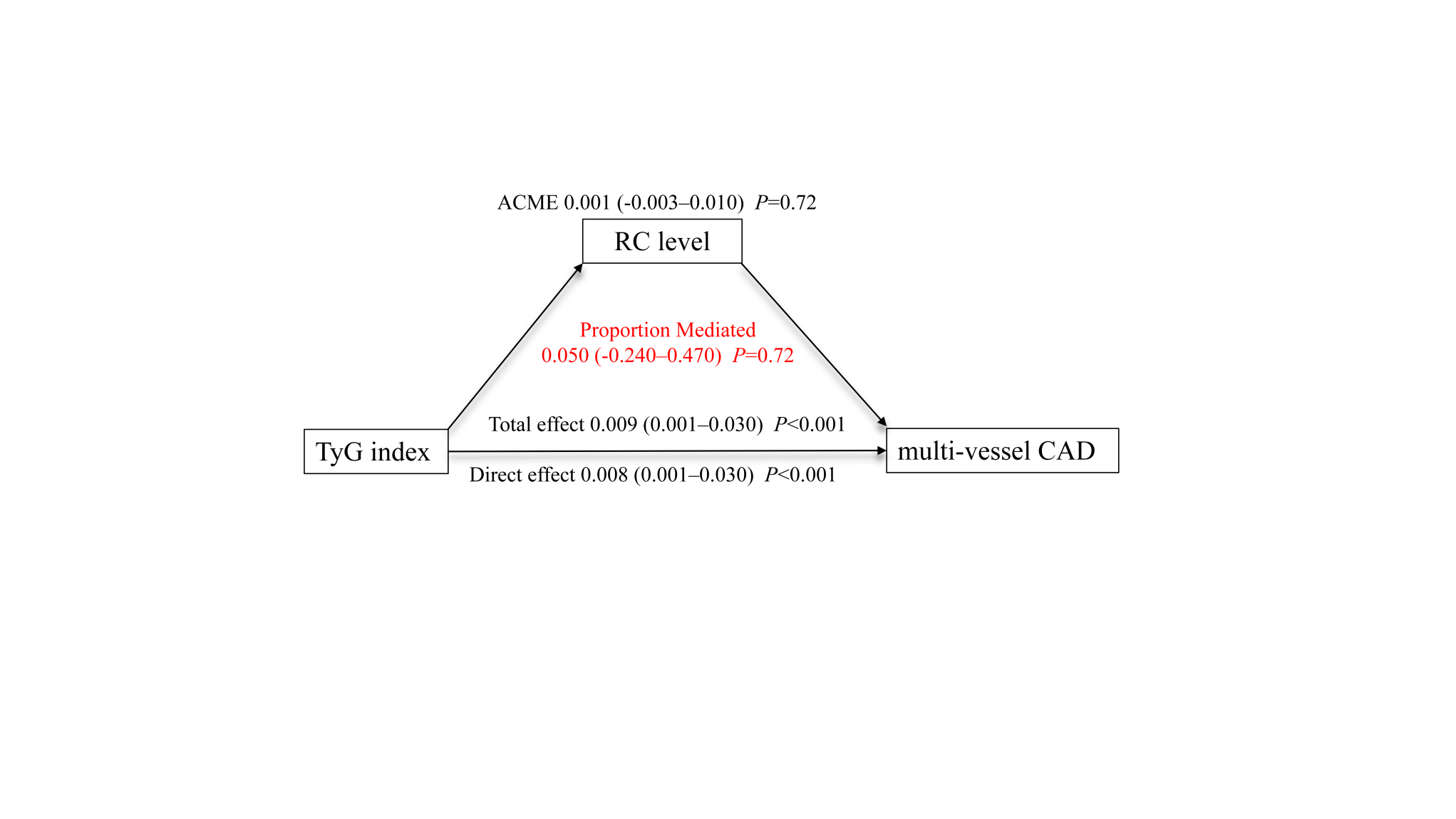


**Supplementary Figure S3** Mediated effects by RC levels on the associations of TyG index with multi-vessel CAD. All models were adjusted for age, gender, smoking status, SBP, hyperlipemia, stroke, AST, albumin, antiplatelet drugs and LVEF. TyG index, triglyceride-glucose index; RC, remnant cholesterol; CAD, coronary artery disease;OR, odds ratios; CI, confidence interval; SBP, systolic blood pressure; AST, aspartate aminotransferase; LVEF, Left ventricular ejection fraction.
